# Anti-inflammatory effect of ApoE23 on *Salmonella typhimurium*-induced sepsis in mice

**DOI:** 10.1515/med-2023-0767

**Published:** 2023-07-25

**Authors:** Chuanqing Wang, Lijun Yin, Pan Fu, Guoping Lu, Xiaowen Zhai, Changsheng Yang

**Affiliations:** Department of Nosocomial Infection Control and the Clinical Microbiology Laboratory, Children’s Hospital of Fudan University, Shanghai 200032, China; Department of Nosocomial Infection Control, Children’s Hospital of Fudan University, Shanghai 200032, China; Department of the Clinical Microbiology Laboratory, Children’s Hospital of Fudan University, Shanghai 200032, China; Department of Pediatric Intensive Care Unit, Children’s Hospital of Fudan University, Shanghai 200032, China; Department of Hematology, Children’s Hospital of Fudan University, Shanghai, 399 Wanyuan Road, Shanghai 200032, China; The Institute of Cardiovascular Diseases of Shanghai, Key Laboratory of Viral Heart Diseases, Ministry of Health, Zhongshan Hospital of Fudan University, 180 Fenglin Road, Shanghai 200032, China

**Keywords:** apoE23, sepsis, *Salmonella typhimurium*, LPS, LDLR, LRP

## Abstract

Two independent experiments were performed with three groups each (sepsis control, sepsis, and sepsis with apoE23 treatment) to investigate the anti-inflammatory effect of apolipoprotein 23 (apoE23) in a mouse model of sepsis induced by *S. typhimurium*. Survival rates; plasma level variations in tumor necrosis factor (TNF)-α, interleukin (IL)-6, and lipopolysaccharide (LPS); S. *typhimurium* colony-forming units in the spleen tissue; and mRNA and protein expression levels of low-density lipoprotein receptor (LDLR), LDLR-related protein (LRP), syndecan-1, and scavenger receptor B1 were evaluated in the livers of mice from the three groups. Results found that the survival rate of septic mice treated with apoE23 was 100% within 48 h, while it was only 40% in septic mice without apoE23 treatment (*P* < 0.001). The plasma LPS, TNF-α, and IL-6 levels and the *S. typhimurium* load in mice in the apoE23-treated group were significantly lower than those in septic mice (*P* < 0.05). Moreover, apoE23 restored the downregulated expression of LDLR and LRP in the liver tissue of septic mice. So apoE23 exhibits an anti-inflammatory effect in the mouse model of *S. typhimurium*-induced sepsis. Further studies are required to understand the mechanisms underlying the anti-inflammatory effects of apoE23.

## 1 Introduction

Sepsis, a life-threatening condition caused by a dysregulated host response to infection, remains the leading cause of death in intensive care unit patients and has been a global health priority since 2017 [1,2]. The hyperactive inflammatory response mediated by immune cells against infectious organisms and their toxins leads to host cell death and tissue damage, which are characteristic of septic shock [2]. Lipopolysaccharide (LPS), also known as endotoxin, is considered the principal trigger of the immune response leading to sepsis [3].

Apolipoprotein E (apoE) is a plasma apolipoprotein with multiple biological functions [4,5,6]. Exogenously, apoE significantly suppresses the production of interleukin (IL)-6, IL-1β, and tumor necrosis factor (TNF)-α induced by LPS in RAW 264.7 cells [4]. ApoE knockout mice are highly susceptible to endotoxemia and *Klebsiella pneumoniae* infection due to their inability to neutralize LPS [5]. These biological functions of apoE are mediated by the low-density lipoprotein receptor (LDLR) family, including LDLR, LDLR-related protein (LRP), syndecans, heparan sulfate proteoglycans (HSPGs), and scavenger receptor B1 (SRB1) [6,7]. Most of these receptors are expressed in hepatic cells and macrophages [6,7].

ApoE23 is a mimetic peptide of apoE that downregulates TNF-α, IL-6, and IL-10 expression in LPS-induced cells and human peripheral blood mononuclear cells [8]. In this study, the effects of intraperitoneal injection of *S. typhimurium*, a gram-negative intracellular pathogen, were studied, along with the therapeutic effect of apoE23 on the *S. typhimurium*-infected mice.

## 2 Materials and methods

### 2.1 ApoE23 synthesis and purification

ApoE23, consisting of amino acid residues 141–148 and 135–149, was synthesized by solid-phase synthesis using standard fluorenylmethoxycarbonyl chemistry protocols. The peptide sequence LRKLRKRLVRLASHLRKLRKRLL was obtained after high-pressure liquid chromatography (>95% purity). A filter-sterilized aqueous solution of apoE23 at a concentration of 0.3 μg/L was prepared before use.

### 2.2 Murine model setting

Forty-five clean level C57BL female mice (2 weeks old after weaning, 10–12 g body weight) were obtained from the Laboratory Animal Research Institute of Shanghai Medical College, Fudan University. *S. typhimurium* colonies remaining on the culture dish were used for the experiment. The strains were identified using MALDI-TOF biotyper mass spectrometry (Bruker Company, Germany). Antimicrobial susceptibility tests were performed using automatic Vitek2 compact machines. The quality control strain was *Escherichia coli* ATCC25922; *S. typhimurium* diagnostic serum was obtained from Ningbo Tianrun Biotechnology Co. Ltd.

For the animal model tests, the mice were randomly divided into three groups of 15 mice each. All mice in the three groups were housed in a laminar flow environment with a 12 h light/dark cycle, a stable temperature of 25°C, and free access to diet and water. Mice survival rates were monitored every 4 h for a total of 72 h. The three model groups of mice were as follows: sepsis apoE23-treated group – infected with 100 μL *S. typhimurium* (1.0 × 10^6^ colony-forming units [CFUs]) [9] by intraperitoneal injection and immediately treated with a single dose of apoE23 (6 mg/kg body weight) [10] by tail vein injection; sepsis group – s infected with the same dose of *S. typhimurium* and immediately treated with 0.9% sodium chloride by tail vein injection; and the sepsis control group – intraperitoneal injection with the same dose of 0.9% sodium chloride, and immediately treated with 0.9% sodium chloride by tail vein injection.

All animals were anesthetized with ether prior to intraperitoneal injection and euthanized with CO_2_ (flow rate: 30% volume displacement per min) after 72 h.

The humane endpoints for euthanizing animals were as follows:

1. Weight loss: When the weight loss reaches 20–25%, or the animals have cachexia or consumptive symptoms.
2. Loss of appetite: Complete loss of appetite for 24 h or poor appetite (less than 50% of the normal amount) for 3 days.
3. Weakness: Inability to eat or drink, animals unable to stand, or those that could only stand with extreme reluctance for 24 h without anesthesia or sedation.
4. Infection

### 2.3 Blood and tissue sample preparation

To observe the mechanism of the therapeutic effect of apoE23, 45 C57BL mice were divided into three groups. The interventions were the same as those mentioned above. After the intervention, the mice were euthanized by CO_2_ at 1, 3, and 24 h (*n* = 5 at each time point). Blood samples were collected from the tail vein in EDTA anticoagulant tubes, centrifuged at 3,000 rpm for 10 min, and stored at −20°C for the TNF-α, IL-6, and LPS assays. Whole blood samples were cultured overnight on blood agar plates to verify bacteremia. Sepsis was confirmed by positive blood cultures and LPS measurements (described below). The lungs, small intestine, and liver were fixed in 10% formalin, paraffin-embedded, and stained with hematoxylin and eosin (HE) for histopathological observation. Pathological changes were observed under a microscope (100× magnification; HE stain). The liver was sectioned into two: the first section was used for histopathological observation (as mentioned above), and the second section was snap-frozen in liquid nitrogen, pulverized on dry ice, and prepared for either quantitative mRNA or protein analysis. The Knodell pathological score [11] was used for the liver pathological score.

### 2.4 *Salmonella typhimurium* CFU analysis in mouse spleen tissue

For direct culture, 10 μL of tissue homogenate from the mouse spleen was incubated in a xylose lysine deoxycholate medium (Chromagar, Shanghai, China). The number of bacterial colonies was calculated and identified using MALDI-TOF mass spectrometry.

### 2.5 Assays for plasma TNF-α, IL-6, and LPS levels

Plasma IL-6 and TNF-α levels were measured using murine IL-6 and TNF-α enzyme-linked immunosorbent assay kits (R&D Co.), respectively. LPS was measured in mouse plasma samples using the dynamic immunoturbidimetric assay with the gram-negative endotoxin determination reagents kit (EKT109; Gold Mountain River Tech, Shanghai, China) and an auto-analyzer (MB-80; Goldstream, Shanghai, China), according to the manufacturer’s instructions. Negative and blank controls were used for each panel.

### 2.6 Quantitative real-time polymerase chain reaction (qRT-PCR) and western blot

To determine the possible mechanism of the anti-inflammatory effect of apoE23, the expression levels of the apoE receptors LDLR, LRP, syndecan-1 (SDC1), and SRB1 in mouse liver were detected using RT-PCR and western blotting; previously described experimental methods were followed [9]. Hepatic cells from mice were lysed, and total RNA was extracted using the TRIzol Max kit (Invitrogen, USA). RNA was reverse transcribed to cDNA using Moloney murine leukemia virus reverse transcriptase (Promega, Madison, WI, USA). The mRNA expression of LDLR, LRP, SDC1, SRB1, and β-actin was determined by qPCR using SYBR Premix Ex Taq (Takara, Dalian, China). β-actin was used as an endogenous control for sample normalization. The following gene-specific primers, noted below, were designed and synthesized by Sangon Biotech, Inc. (China).

LDLR-F: 5′-CCGACCTGATGAATTCCAGT-3′;

LDLR-R: 5′-TGGTCTTGCACTCCTTGATG-3′.

LRP-F: 5′-CGACACCAACAAGAAGCAGA-3′;

LRP-R: 5′-AGAGTGTGGTTGCTCCCATC-3′.

SDC1-F: 5′-TGCGTACAACAGGGTATGGA-3′;

SDC1-R: 5′-CCTCCCCTCCACTCCTAGAC-3′.

SRB1-F: 5′-GGGCTCGATATTGATGGAGA-3′;

SRB1-R: 5′-GGAAGCATGTCTGGGAGGTA-3′.

β-actin-F: 5′-GAGACCTTCAACACCCCAGC-3′;

β-actin-R: 5′-ATGTCACGCACGATTTCCC-3′.

The PCR thermal cycling program consisted of one cycle at 95°C for 30 s, followed by 40 cycles at 95°C for 5 s and 60°C for 30 s. A melting curve was generated by setting the cycles at 95°C for 15 s, 60°C for 30 s, and 95°C for 15 s. The confirmation of a single gene product was achieved by generating a dissociation curve after each qPCR cycle. The cycle threshold value was determined using iCycler software, and quantification of gene products, normalized to the expression of the ribosomal β-actin housekeeping gene, was calculated using the comparative Ct (2^−ΔΔCt^) method.

### 2.7 Western blot

The total hepatic cell protein levels were assayed using a BCA Protein Assay kit (Beyotime, Shanghai, China). Lysates (50 μg total protein) were mixed with 5× sample buffer, heated to 100°C for 5 min, and separated using 10% sodium dodecyl-sulfate polyacrylamide gel electrophoresis. The resolved proteins were transferred onto a 0.45 μm PVDF membrane using a Mini-Protean 3 electrophoresis system (Bio-Rad, USA). Non-specific binding sites on the membranes were blocked by incubation with a buffer containing 5% (w/v) non-fat milk. The membranes were probed with primary rabbit anti-mouse antibodies against *LDLR* (1:2,500; Epitomics, Burlingame, USA), *LRP* (1:2,500; Epitomics, Burlingame, USA), *SDC1* (1:1,000; Abcam, Cambridge, USA), *SRB1* (1:5,000; Abcam, Cambridge, USA), and β-actin (1:3,000; Santa Cruz Biotechnologies, Santa Cruz, USA). Immunoreactive bands were detected by incubation with horseradish peroxidase-conjugated secondary antibodies (1:3,000; Santa Cruz Biotechnologies, Santa Cruz, USA) and visualized by chemiluminescence. Protein bands were quantified using the Quantity One software (Bio-Rad, Hercules, USA) and normalized to the corresponding β-actin bands.

### 2.8 Statistical methods

The detection values are expressed as the mean value ± standard deviation (SD). Analysis of variance was used to compare the mean of detection values between groups, and Tukey’s Test was used as a post hoc test. Survival curves were generated using the Kaplan–Meier method, and survival differences were analyzed using the log-rank test. Statistical significance was set at *P* < 0.05. SPSS 25.0 was used for all statistical analyses.


**Ethics statement:** This study was approved by the Ethical Committee on Animal Experiments at the Children’s Hospital of Fudan University (approval number (2011) 023).

## 3 Results

### 3.1 Cumulative survival rates in the septic mice

**Figure 1:**
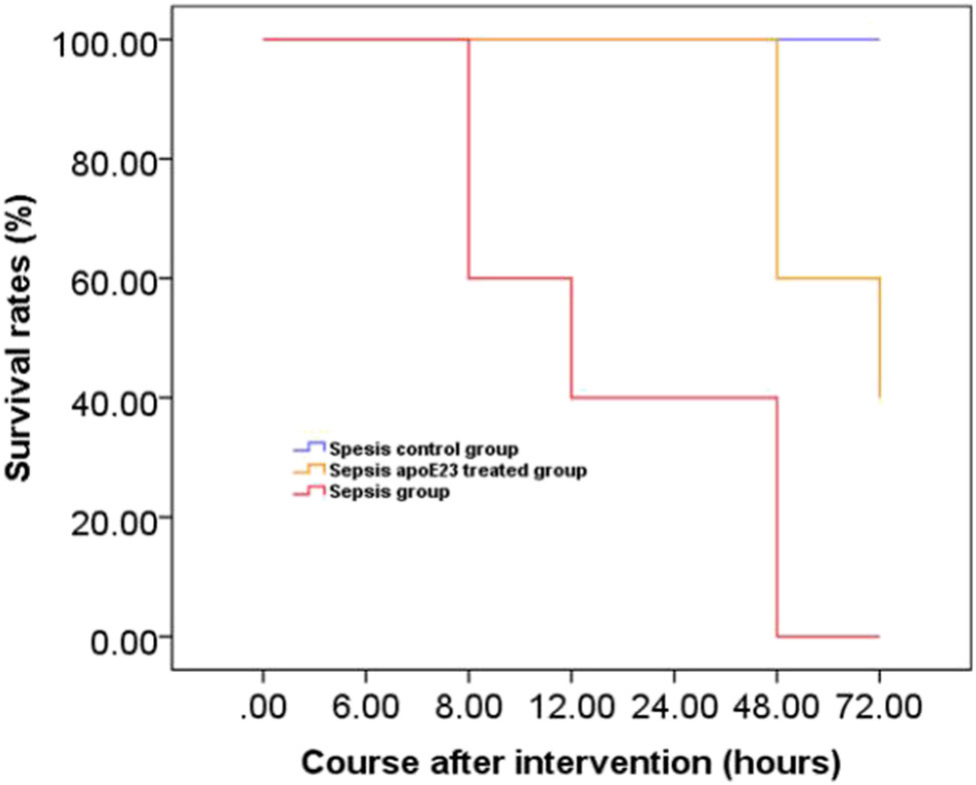
Kaplan–Meier curve of cumulative survival rate between groups ApoE23 improved the overall survival rate of mice with sepsis. The mice were randomly divided into three groups (the sepsis control, sepsis, and sepsis apoE23 treated group) of 15 and mice survival rates were observed every 4 h for 72 h.

The survival rates of mice from different groups were evaluated to investigate whether apoE23 could improve their survival rate. No mice in the sepsis control group died during the observation period. The median survival time of mice in the sepsis group was 24 h and the cumulative survival rate of mice in the sepsis group that were treated with apoE23 was 60% at 48 h (median survival time was not calculated) (*P* < 0.001) (Figure 1). Therefore, apoE23 improved the overall survival rate of mice. However, since there were only five mice in each group at each time point, a larger sample size is required to determine whether apoE23 delays death.

### 3.2 ApoE23 attenuated infection-induced organ injury

**Figure 2:**
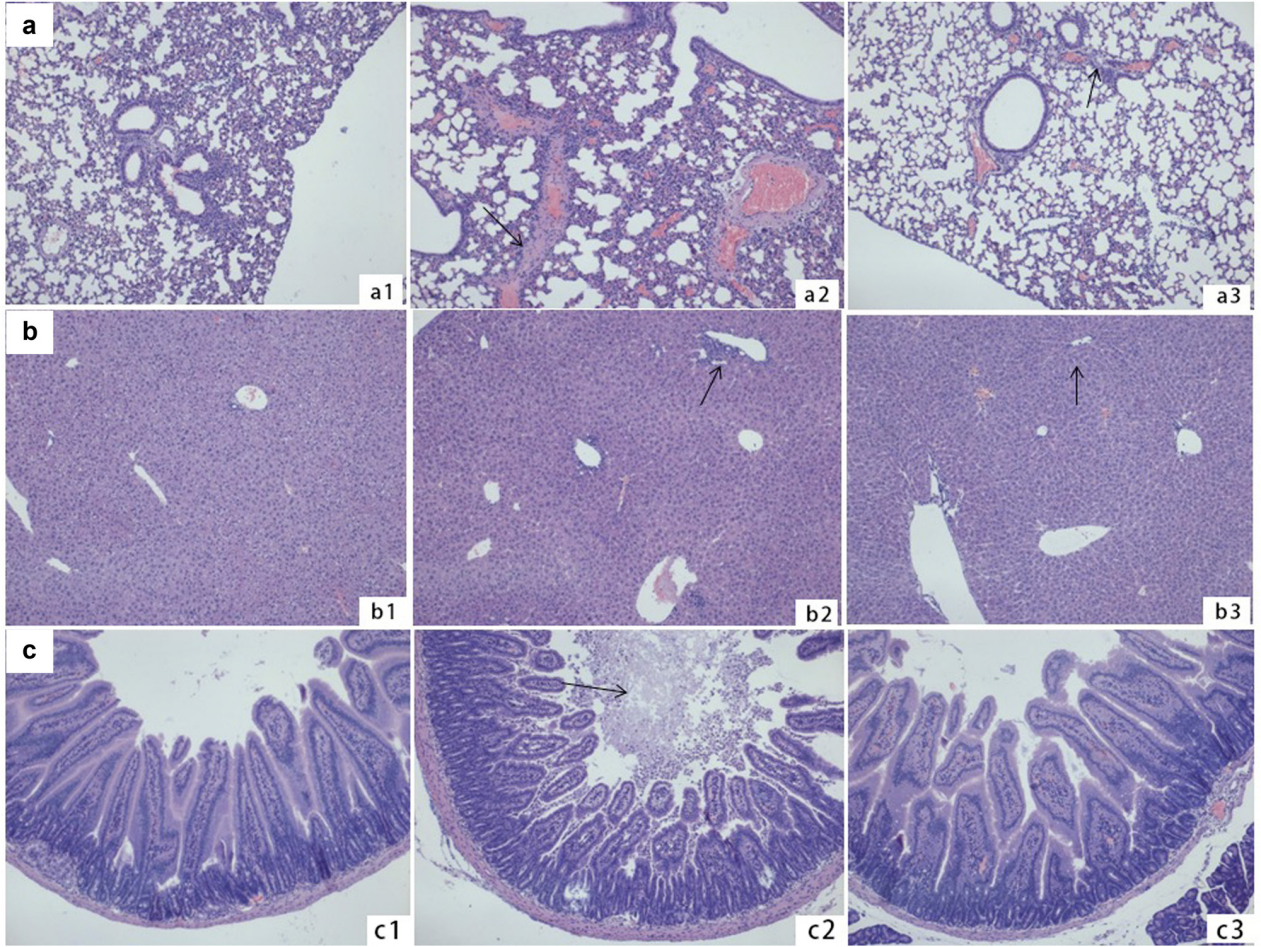
ApoE23 attenuated infection-induced lung, small intestine, and liver histopathological injury. Mice were euthanized at 1, 3, and 24 h (*n* = 5 for each time point) after tail vein treatment. Histopathological sections were stained with hematoxylin and eosin for histopathological observation. (a) Histopathological sections of lungs tissues. a1: The sepsis control group; the alveoli were intact and well filled, without inflammatory cell exudation. a2: The sepsis group; many inflammatory cells infiltrated around the pulmonary vessels. a3: The sepsis apoE23 treated group; the alveoli were intact and well filled, with a small amount of focal inflammatory cells exuding. (b) Histopathological sections of the liver tissue. b1: The sepsis control group; no hemorrhage or infiltration of inflammatory cells were found in the liver tissue. b2: The sepsis group; showed significant infiltration of inflammatory cells around the portal area. b3: The sepsis apoE23 treated group; there was mild inflammatory cell infiltration around the liver sinusoids. (c) Histopathological section of the small intestine. c1: In sepsis control mice, the intestinal villi were intact, and there was no inflammatory secretion in the intestinal cavity. c2: the sepsis group; a large amount of inflammatory cells exudation was found in the intestinal cavity. c3: the sepsis apoE23 treated mice; a small amount of inflammatory exudation was found in the small intestine cavity.

Histopathological sections of the lungs, liver, and small intestine of mice in the three groups were stained with HE and observed under an optical microscope to assess histopathological injury in the organs. The lung tissue in the sepsis group was slightly edematous, and many inflammatory cells had infiltrated around the pulmonary vessels (Figure 2, a2). In addition, inflammatory cell exudation was observed in the liver (Figure 2, b2). The murine small intestinal cavities were filled with inflammatory exudate in the sepsis group (Figure 2, c2). In contrast, only slight inflammatory injuries were observed in apoE23-treated septic mice (Figure 2a3, b3, and c3).

These histopathological changes indicated that *S. typhimurium* infection induced prominent inflammatory injuries in various organs of the mice, while apoE23 treatment minimized the inflammatory injury. However, the Knodell pathological score showed only a few inflammatory cells around the portal area, and no degeneration or necrosis was found in the liver lobule. The pathological grading of mouse liver showed mild changes both in the septic and apoE23-treated septic mice within 24 h (score 1). Longer observations after 24 h are required to monitor the anti-inflammatory effect of apoE23.

### 3.3 ApoE23 reduced bacterial load in septic mice

**Figure 3:**
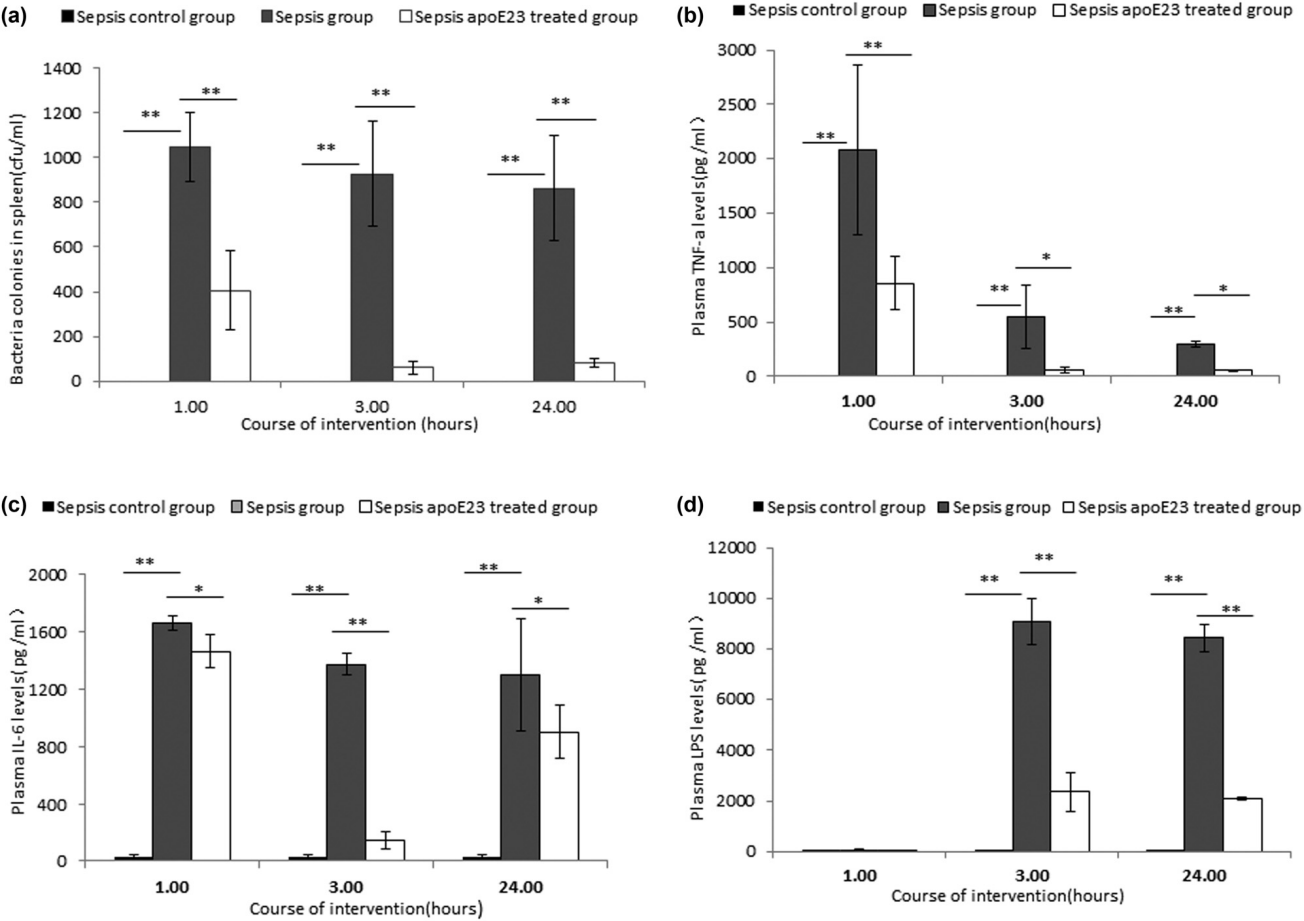
ApoE23 treatment reduced bacterial colonies in (a) spleen tissue, (b) decreased plasma TNF-α, (c) IL-6, and (d) LPS levels in the sepsis group. The number of bacterial colonies in the spleen was calculated and identified using matrix-assisted laser desorption/ionization time-of-flight mass spectrometry. Plasma levels of IL-6 and TNF-α were measured by ELISA, and LPS was measured using a dynamic immunoturbidimetric assay. Results are presented as the mean value ± standard deviation (SD). *P < 0.05; **P < 0.01.

The number of bacterial colonies in the spleen tissue homogenate from mice in different groups was evaluated (Figure 3). No bacteria were isolated from mice in the sepsis control group. The bacterial colonies in the spleen tissue homogenate in the sepsis group increased by more than 800 CFU/mL at the three time points. After apoE23 treatment, the number of bacterial colonies dramatically decreased (1,048 ± 152 vs 405 ± 178 CFU/mL [*P* < 0.01] at 1 h, 926 ± 236 vs 59 ± 29 CFU/mL [*P* < 0.01] at 3 h, and 862 ± 235 vs 82 ± 22 CFU/mL [*P* < 0.01] at 24 h) (Figure 3a). These results indicate that apoE23 has a dramatic bactericidal effect *in vivo*.

### 3.4 ApoE23 reduced plasma TNF-α, IL-6, and LPS levels in septic mice

Plasma TNF-α, IL-6, and LPS levels in mice from different groups were evaluated to investigate whether apoE23 has an effect on their levels (Figure 3).

The plasma TNF-α level in the sepsis group increased dramatically at the 1 h time point and then continued to decrease at 3 h and 24 h compared to the sepsis control group (*P* < 0.01). Plasma IL-6 levels at the three time points were higher in the sepsis group than in the control group (*P* < 0.01). ApoE23 treatment dramatically decreased plasma TNF-α and IL-6 levels at the three time points compared to those in the sepsis group (Figures 3b and 3c). The plasma LPS levels increased dramatically at the 3 h and 24 h time points in the sepsis group compared with those in the sepsis control group (*P* < 0.01). ApoE23 treatment dramatically decreased plasma LPS levels at the 3 h and 24 h time points compared to the sepsis group (*P* < 0.05) (Figure 3d) while no significant differences were found between these two groups at the 1 h time point.

These results indicate that apoE23 can downregulate the plasma TNF-α, IL-6, and LPS levels in the septic mouse and exert anti-inflammatory effects.

### 3.5 Effect of apoE23 on the expression levels of apoE-related receptors in livers of septic mice

**Figure 4:**
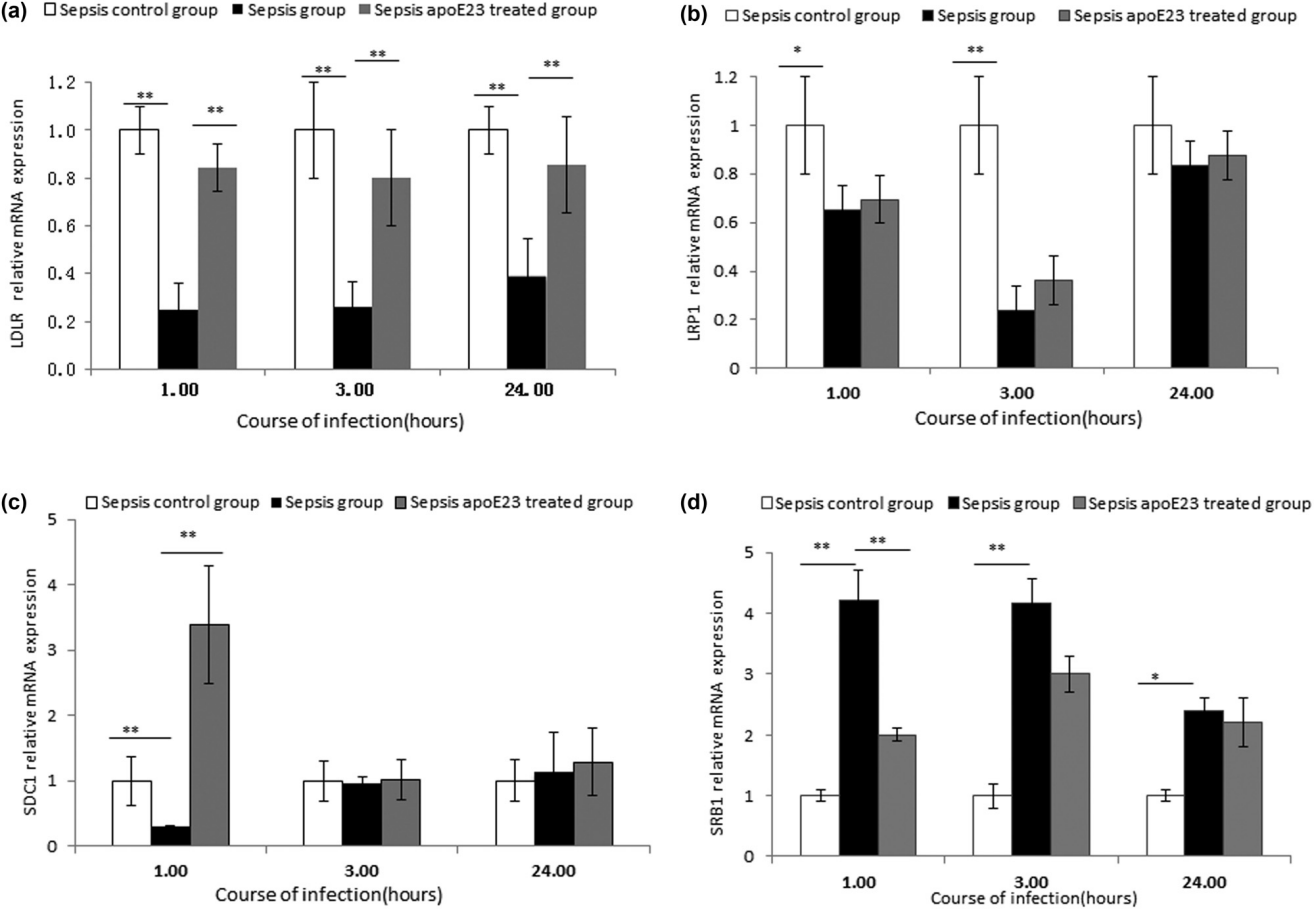
ApoE23 restored the mRNA expression of LDLR, LRP, SDC1, and SRB1 to different degrees at different times in the liver. Hepatic mRNA expression levels of (a) LDLR, (b) LRP, (c) SDC1, and (d) SRB1 were detected using qPCR. Expression levels were normalized to β-actin. *P < 0.05; **P < 0.01.

**Figure 5:**
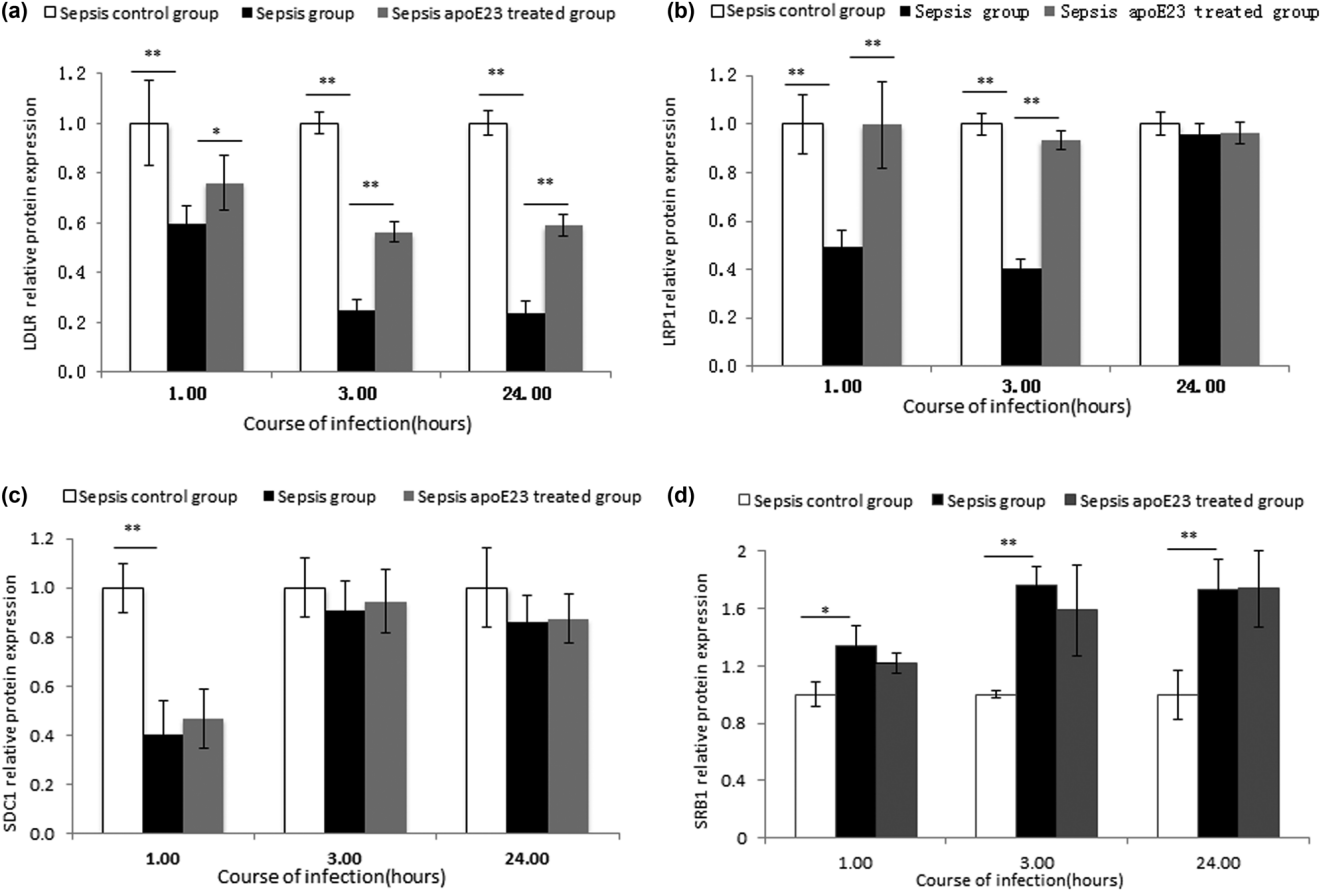
ApoE23 restored the protein expression of LDLR, LRP, SDC1, and SRB1 to different degrees at different times in the liver. Hepatic (a) LDLR, (b) LRP, (c) SDC1, and (d) SRB1 levels were measured by western blotting. The expression levels were normalized to GAPDH. *P < 0.05; **P < 0.01.

The expression levels of LDLR, LRP, SDC1, and SRB1 relative to those of β-actin in the liver were evaluated at the transcriptional and translational levels to investigate the relationship between the anti-inflammatory effect of apoE23 and the expression of these LPS-related receptors (Figures 4 and 5, respectively).

Compared to the sepsis control group, LDLR expression in the sepsis group decreased significantly at the three time points (*P* < 0.01), while LRP expression decreased at 1 h and 3 h (*P* < 0.05), both at the transcriptional and translational levels. SDC1 expression only decreased at the transcription level at 1 h (*P* < 0.01).

Compared to the sepsis group, LDLR expression was significantly recovered at the three time points in both the transcription and translation levels in the sepsis apoE23 treated group (*P* < 0.01). No significant differences were found in LRP mRNA levels, whereas the LRP protein levels at the 1 and 3 h time points were significantly recovered in the sepsis apoE23 treated group (*P* < 0.05). Only a transient increase at 1 h in the SDC1 mRNA levels was detected in the sepsis apoE23 treated group (*P* < 0.01) (Figures 4 and 5).

Unlike LDLR, LRP, and SDC1, SRB1 expression increased significantly at the 3 h time point in both the transcription and translation levels in the sepsis group compared to the sepsis control group (*P* < 0.01) (Figure 4d). With apoE23 treatment, no significant difference in SRB1 expression was found, except for the transcription levels that decreased at the 1 h time point in the sepsis apoE23 treated group (*P* < 0.01) (Figures 4 and 5).

These results indicate that apoE23 has an effect on LDLR, LRP, SDC1, and SRB1 at different stages of sepsis.

## 4 Discussion

The study sought to examine the anti-inflammatory impact of apoE23 in a mouse model of *S. typhimurium*-induced sepsis. It revealed that apoE23 therapy effectively reduced plasma levels of TNF-α, IL-6, and LPS; decreased bacterial load in the spleen tissue homogenate; and alleviated infection-induced lung, liver, and small intestine injuries in mice with sepsis. These findings suggest that the anti-inflammatory effect of apoE23 may be attributed to the upregulation of apoE-related receptors, specifically LDLR and LRP.

Although several animal models of sepsis have been established, none perfectly replicate all clinical manifestations and pathophysiological changes observed in clinical sepsis [12,13]. In this study, an *S. typhimurium* infection-induced murine sepsis model was established based on the ability of the zoonotic bacterium to cause severe invasive infection in mice [14] and the similarity of the pathological process of sepsis or septic shock in *S. typhimurium*-infected mice to the clinical conditions [15,16,17]. Injecting bacteria offers advantages for observing drug efficacy and studying bacterial or LPS clearance hemodynamics [12,13]. Lipoproteins, including apoE, are known to play a key role in downregulating systemic inflammation in preclinical sepsis models [18,19,20] and facilitating the clearance of gram-negative bacteria by binding to and neutralizing LPS [21].

Among the plasma lipoproteins that are important host defense factors against *S. typhimurium* infection [22], apoE has been closely associated with sepsis. ApoE protects against bacterial LPS-induced lethality, and recombinant apoE shows potential therapeutic application in protecting against LPS-induced endotoxemia [23]. Several apoE-mimetic peptides have been developed based on the sequence of 1–149 amino acid residues in the N domain of apoE and have demonstrated their efficacy against inflammation, both *in vitro* and *in vivo* [24,25,26]. In this study, all the experimental mice exhibited septic-shock-like manifestations and died within 24 h after being infected with a half-lethal dose of *S. typhimurium*, whereas with a single dose of apoE23 treatment, the mortality of the septic mice declined. A previous study also found that ApoE23 downregulates TNF-α and IL-6 expression in LPS-induced cells and human peripheral blood mononuclear cells [8]. Further investigation demonstrated that apoE23 reduced plasma TNF-α, IL-6, and LPS levels; decreased bacterial load in spleen tissue; and attenuated infection-induced lung, liver, and small intestine injuries in septic mice. The observations suggest that the efficacy of the apoE-mimetic peptides in attenuating pro-inflammatory cytokine production could be attributed to the neutralization of plasma LPS, the blocking of LPS binding to macrophages, and the direct downregulation of pro-inflammatory cytokine expression.

The absorption of the LPS complex is mediated by the binding of the apoE LDLR domain with LDLR, LRP, HSPG (SCD1), and SRB1 expressed in hepatic cells [6,7,27,28,29]. ApoE has two distinct functional domains, and the motif includes the receptor-binding region of apoE [6]. This region, enriched in basic residues, is responsible for high-affinity ligand binding to the LDL superfamily of receptors. Residues 142–147 within this sequence, known as the heparin-binding domain, mediate the attachment of apoE to cellular HSPGs [30,31]. Although tandem-repeat peptides derived from the receptor-binding region also bind to the LRP [32], their binding is a complex process that likely requires the participation of HSPGs [33]. This signal cascade is activated when lactoferrin, lipoprotein lipase, or Pseudomonasexotoxin A binds to LRP; however, binding of LDL or receptor-associated protein to LRP fails to trigger signal transduction [34,35].

LDLR is the most crucial receptor of apoE. The combination of apoE and LDLR receptors facilitates the removal of apoE glycoproteins, chylomicrons, and high-density lipoproteins. LDLR also exhibits anti-inflammatory properties. Interferents that enhance LDL clearance or increase LDLR expression may reduce endotoxemia and protect against severe sepsis [25]. The expression of LDLR in hepatic cells is attenuated during sepsis [26]. In contrast, higher expression was observed in liver macrophages [24]. This imbalance in LDLR expression in the septic mouse liver leads to competitive binding of LPS to macrophages. This redistribution of LPS kinetics not only attenuates the efficacy of LPS clearance by hepatic cells but also triggers the expression of pro-inflammatory cytokines in macrophages [24]. This study corroborated the variation in LDLR expression in septic mice and demonstrated that apoE23 treatment can reverse the downregulation of LDLR expression. These results indicate that LDLR functionally contributes to all stages of sepsis development and that apoE23 has a regulatory effect throughout the process.

LRP, another important receptor in the LDLR family, is associated with over 40 different ligands, including lipoproteins, proteases, protease inhibitor complexes, bacterial toxins, viruses, and various intracellular proteins. These ligands activate functions of LRP, such as maintaining the stability between proteases and protease inhibitors, resistance to viral and toxin invasion, regulation of lysosomal enzyme activation, and anti-inflammatory effects [36]. The study results revealed a decline in LRP expression at the transcriptional and translational levels during the early stages of sepsis. Following bacterial inoculation, hepatic LRP expression decreased specifically at the 1 and 3 h time points, but not at the 24 h time point, which represents the late stage of sepsis. The sepsis-induced downregulation of LRP protein levels was significantly recovered at 1 and 3 h after apoE23 treatment at the translation level. Additionally, SDC1 is a predominant component of HSPGs and can independently regulate lipid metabolism without affecting the LDLR family [37]. SDC1 appears to be involved only in the early stage (1 h time point) of sepsis in mice and transiently recovered after apoE23 treatment. The shedding of SDC1 from human hepatocytes alters very-low density lipoprotein clearance [38]. Further investigation is required to determine whether the changes in LRP and SDC1 expression affect the metabolism of the LPS-apoE lipoprotein complex. Unlike LDLR, LRP, and SCD1 receptors, SRB1 expression increased rapidly in response to bacterial injection at the three time points but decreased transiently after apoE23 treatment. SRB1 is a high-density lipoprotein receptor that is expressed predominantly in the liver [39]. It binds to and neutralizes LPS, contributing to its anti-inflammatory effect [40]. The effect of apoE23 on SRB1 was transient or unclear, mainly because free apoE or apoE mimetic peptides, such as apoE23, preferentially bind to lipids to promote lipid absorption, thereby competitively weakening the apoE-SRB1 anti-inflammatory effect [41].

The study has several limitations. First, the sample size was small. Second, only female mice were used in the experiments, and the phase of the estrous cycle was not considered. Additionally, while abnormal pathological changes were evident in HE staining, other staining data, such as collagen deposition or fibrosis-related staining, would provide a comprehensive assessment of the inflammatory damage. Moreover, a separate treatment group of apoE for normal mice was not included, preventing the determination of potential side effects of apoE23 on normal mice. Finally, while the study illustrated the anti-inflammatory effect of apoE23 on *S. typhimurium*-induced sepsis in mice, further research is needed to understand the mechanisms and signaling pathways involved in this effect.

The study findings indicated that sepsis-induced changes in the expression levels of apoE-related receptors in the liver are likely to be an acute response to infection. Following apoE23 treatment, the sepsis-induced downregulation of LDLR and LRP was significantly restored. However, the effect of apoE23 on SDC1 and SRB1 expression remains transient or unclear.

## 5 Conclusion

The investigation revealed that apoE23 therapy effectively mitigates sepsis with its direct bioactivity against intracellular bacteria and its anti-endotoxemia effect. Furthermore, apoE23 appears to modulate LDLR expression in both the early and late stages of sepsis, while its effect on LRP, SDC1, and SRB1 was only observed in the early stage. Further research is required to fully understand the effects of apoE23 on apoE-related receptors.
